# All-cause mortality and risk factors in a cohort of retired military male veterans, Xi'an, China: an 18-year follow up study

**DOI:** 10.1186/1471-2458-7-290

**Published:** 2007-10-12

**Authors:** Xiao Y Sai, Yao He, Ke Men, Bo Wang, Jiu Y Huang, Qiu L Shi, Lei Zhang, Liang S Li, Bernard CK Choi, Yong P Yan

**Affiliations:** 1 Department of Epidemiology, College of Military Services and Statistics, Fourth Military Medical University, Xi'an, China; 2 Department of Epidemiology, Institute of Geriatrics, Chinese PLA General Hospital, Beijing, China; 3 Department of Public Health Sciences, University of Toronto, Toronto, Canada; 4 Department of Epidemiology and Community Medicine, University of Ottawa, Ottawa, Canada

## Abstract

**Background:**

Risk factors of all-cause mortality have not been reported in Chinese retired military veterans. The objective of the study was to examine the risk factors and proportional mortality in a Chinese retired military male cohort.

**Methods:**

A total of 1268 retired military men aged 55 or older were examined physically and interviewed using a standard questionnaire in 1987. The cohort was followed up every two years and the study censored date was June30, 2005 with a follow-up of up to 18 years. Death certificates were obtained from hospitals and verified by two senior doctors. Data were entered (double entry) by Foxbase, and analysis was carried out by SAS for Windows 8.2. Multivariate Cox proportional hazard regression model was used to compute hazard ratio (HR) and 95% confidence interval (CI).

**Results:**

The total person-years of follow-up was 18766.28. Of the initial cohort of 1268 men, 491 had died, 748 were alive and 29 were lost to follow up. Adjusted mortality (adjusted for age, blood pressure, body mass index, cholesterol, triglycerides, alcohol, exercise, and existing disease) was 2,616 per 100,000 person years. The proportional mortality of cancer, vascular disease and Chronic Obstructive Pulmonary Disease (COPD) were 39.71%, 28.10% and 16.90% respectively. Multivariate analysis showed that age, cigarettes per day, systolic blood pressure, triglyceride, family history of diseases (hypertension, stroke and cancer), existing diseases (stroke, diabetes and cancer), body mass index, and age of starting smoking were associated with all-cause mortality, HR (95%CI) was1.083(1.062–1.104), 1.026(1.013–1.039), 1.009(1.003–1.015), 1.002(1.001–1.003), 1.330(1.005–1.759), 1.330(1.005–1.759), 1.444(1.103–1.890), 2.237(1.244–4.022), 1.462(1.042–2.051), 2.079(1.051–4.115), 0.963(0.931–0.996)and 0.988(0.978–0.999)respectively. Compared with never-smokers, current smokers had increased risks of total mortality [HR 1.369(1.083–1.731)], CHD [HR 1.805 (1.022–3.188)], and lung cancer [HR 2.939 (1.311–6.585)].

**Conclusion:**

The three leading causes of diseases were cancer, CHD and stroke, and COPD. Aging, cigarette smoking, high systolic blood pressure, high triglyceride, family history of cancer, hypertension and stroke, existing cases recovering from stroke, diabetes and cancer, underweight, younger age of smoking were risk factors for all-cause mortality. Quitting cigarette smoking, maintaining normal blood pressure, triglyceride and weight are effect control strategies to prevent premature mortality in this military cohort.

## Background

Chronic non-communicable diseases were estimated to account for 35 million or 60% of all deaths globally in 2005[1]. It is vital to identify risk factors and control the rapidly growing epidemic of chronic diseases. In China, risk factors for all-cause mortality have not been reported, especially in retired military veterans. Two questions remain to be resolved: one was "which diseases had a huge burden among retired military veterans?" the other was "how to prevent them?" To identify the risk factors for all-cause mortality and to improve the health service for retired military veterans, a Chinese retired military male cohort was set up in 1987 and followed up to June 30, 2005.

## Methods

### Study Population

From February to June in 1987, a cross-sectional survey was carried out in 22 military retirement centers in Xi'an, China. As there were few women, only men (1268 or 98% of all eligible) were included in the survey.

### Baseline Measurements

Each participant was interviewed and completed a standardized questionnaire that included a range of demographic factors, aspects of medical history, family history of diseases, and lifestyle. The questionnaire were designed and defined using the WHO MONICA criteria [2]. The physical examinations and interview were carried out by trained nurses and physicians.

The average age of entering the cohort was 62.95 ± 5.18 years. Existing diseases included coronary heart disease (CHD), stroke, hypertension, diabetes, Chronic Obstructive Pulmonary Disease(COPD)and cancer, which were diagnosed by local hospitals. Height was measured in meters (without shoes), and weight in kilogram (with heavy clothing removed and 1 kg deducted for remaining garments). Body mass index (BMI) was calculated as weight (kg)/height (m)^2^. BMI was divided into four levels: <18.5, 18.5–23.9, 24–27.9, and ≥ 28. Total cholesterol and triglycerides were measured using the standard enzymatic method and Hontzschs test (acetylacetone), respectively [3]. Triglycerides was divided into four levels: <90 mg/dl, 90–122 mg/dl, 123–151 mg/dl, and ≥ 152 mg/dl.

Two blood pressure recordings were obtained from the right arm of patients in a sitting position after 30 minutes of rest; measurements were taken in 5-minute intervals, and mean values were calculated. Systolic blood pressure was divided into four levels: <120 mmHg, 120–129 mmHg, 130–139 mmHg, and ≥ 140 mmHg. According to the standard of World Health Organization (WHO) in 1978, borderline hypertensive was defined as one whose systolic blood pressure was between 141~159 mmHg and diastolic pressure was between 91~95 mmHg; Hypertension was defined as one whose systolic blood pressure was higher than 159 mmHg and diastolic pressure was higher than 95 mmHg.

The categories of smoking were never smoking, former smoking, and current smoking. An ever-smoker was defined as one who had smoked at least one cigarette daily for one year or more. Current smokers were ever-smokers who were smoking at baseline, and former smokers were those who had stopped for at least two years. Smoking index = cigarettes per day × duration of smoking. Smoking index was divided into four levels: <350, 350–569, 570–749, and ≥ 750.

Current drinking was defined as average alcohol consumption of more than 10 g of absolute alcohol per day for more than 1 year in the past 5 years.

### Follow-up

The cohort was a fixed cohort which was followed up every two years for all cause death. Death certificates were obtained from hospitals and verified by two senior doctors. The causes were coded according to the International Classification of Diseases, Ninth Edition.

### Statistical Analysis

Data were entered (double entry), and analysis was carried out by SAS for Windows 8.2. Multivariate Cox proportional hazard regression model was used to compute HR and 95% CI. Potential confounders adjusted for included age, systolic blood pressure, BMI, total cholesterol, triglycerides, regular alcohol consumption (yes or no), regular exercise(yes or no), family history of diseases(yes or no) and existing disease(yes or no, or years of disease, as appropriate).

### Ethical considerations

The committee for medical ethics of the Chinese PLA General Hospital approved the study. Each participant signed an informed consent form before completing the questionnaire.

## Results

Up to June 30, 2005, a total of 748 individuals were alive, 29 were transferred away with unknown vital status (the last date of known survival, which was end of Jan 1997, was considered as the censored date), and 491 had died. Adjusted mortality was 2616 per 100,000 person years. Death certificates were available for all the deaths since they all occurred in hospitals. The five leading causes of death were malignant tumor (39.71%), COPD (16.90%), CHD (16.90%), stroke (11.20%) and diabetes (4.68%). Adjusted mortality rates were 1039, 442, 421, 293 and 123 per 100,000 person years respectively.

Table 1 shows characteristics of never, ever smokers at baseline in 1987. Age, BMI, diastolic, systolic, total cholesterol, triglycerides, existing disease including stroke, hypertension, diabetes, COPD and cancers between two groups had no significant differences (P > 0.05).64.89% and 59.20% of ever smokers exercised and drank regularly, which were higher than never smoker(P < 0.05). Prevalence of CHD in ever smokers was higher than never smokers (29.89% vs. 23.71%, P < 0.05).

**Table 1:** Characteristics of never, ever smokers at baseline in 1987

	Never smoker (n = 388)	Ever smoker (n = 880)
Age, mean(SD), years	62.52(5.20)	63.13(5.16)
BMI, mean(SD)	24.36(2.95)	24.31(3.09)
BP, mean(SD), mmHg		
Diastolic	80.60(11.41)	79.72(10.83)
Systolic	129.55(18.86)	129.01(18.12)
Total cholesterol, mg/dl, mean(SD)	193.92(43.84)	196.69(43.04)
Triglycerides, mg/dl, mean(SD)	133.62(72.95)	133.02(65.20)
Duration of follow up, years, mean(SD)	15.23(5.30)	14.61(5.50)
Regular exercises, Yes, n (%)*	221(56.96)	571(64.89)
Regular alcohol, Yes, n (%)*	129(33.25)	521(59.20)
Existing disease(Yes)		
CHD, n (%)*	92(23.71)	263(29.89)
Stroke, n (%)	6(1.55)	14(1.59)
Hypertension, n (%)	87(22.42)	209(23.75)
Diabetes, n (%)	20(5.15)	58(6.59)
COPD, n (%)*	49(12.63)	246(27.95)
Cancers, n (%)	4(1.03)	15(1.70)

* Chi-square, two degrees of freedom: *p *< 0.05

Table 2 shows adjusted HR of major causes of death by smoking status at baseline. Compared with never-smokers, current smokers had increased risks of total mortality [HR 1.369(1.083–1.731)], CHD [HR 1.805 (1.022–3.188)], and lung cancer [HR 2.939 (1.311–6.585)].

**Table 2:** Adjusted^a ^Hazard Ratio of major causes of death by smoking status at baseline

	Never-smoker (N = 388)	Former smoker (N = 461)				Current smoker (N = 419)			
			
	No. of death	No. of death	HR	95%CI	P	No. of death	HR	95%CI	P
All causes	126	193	1.089	0.865–1.372	0.467	172	1.369	1.083–1.731	0.009
CHD	21	25	0.681	0.376–1.233	0.205	33	1.805	1.022–3.188	0.042
Stroke	17	20	0.863	0.447–1.666	0.660	18	1.087	0.552–2.141	0.808
Lung cancer	8	27	2.622	1.165–5.900	0.020	25	2.939	1.311–6.585	0.009
COPD	14	42	1.856	0.993–3.470	0.053	27	1.517	0.784–2.936	0.216

a: Adjusted for age, systolic blood pressure, body mass index, total cholesterol, triglycerides, regular alcohol consumption, exercise, as well as existing disease at baseline.

Table 3 shows that age, cigarettes per day, duration of smoking, systolic blood pressure, triglycerides, negative affairs, exercise, family history of diseases including hypertension, stroke and cancer and existing diseases (including CHD, stroke, hypertension, cerebral arteriosclerosis, diabetes, COPD and cancer) were associated with all cause mortality (P < 0.05). After adjusted for all other factors under study, age, cigarettes per day, systolic blood pressure, triglycerides, BMI, age of starting smoking, family history of diseases including cancer, hypertension and stroke, and existing diseases including stroke, diabetes and cancer were found to be associated with all cause mortality (P < 0.05).

**Table 3:** Hazard Ratios and 95% CI for risk factor for all cause mortality

Risk Factor	Crude HR^1^	95% CI	Adjusted HR^2^	95% CI	P
age(year)	1.091	1.074–1.109	1.083	1.062–1.104	<0.001
systolic blood pressure(mmHg)	1.012	1.007–1.016	1.009	1.003–1.015	0.003
triglycerides(mg/dl)	1.002	1.001–1.003	1.002	1.001–1.003	0.009
Smoking status					
cigarettes per day(number)	1.023	1.015–1.032	1.026	1.013–1.039	<0.001
duration of smoking(year)	1.012	1.006–1.017	1.005	0.997–1.013	0.218
age of starting smoking(year)	1.003	0.996–1.011	0.988	0.978–0.999	0.028
exercise(yes/no)	0.920	0.888–0.953	0.922	0.739–1.152	0.476
BMI(kg/m^2^)	0.986	0.957–1.015	0.963	0.931–0.996	0.026
negative affairs(yes/no)	1.471	1.172–1.845	1.173	0.927–1.483	0.183
family history(yes/no)					
hypertension	1.455	1.122–1.886	1.330	1.005–1.759	0.046
stroke	1.455	1.122–1.886	1.330	1.005–1.759	0.046
cancer	1.282	1.000–1.644	1.444	1.103–1.890	0.007
Existing disease(yes/no)					
cancer	3.596	2.150–6.015	2.079	1.051–4.115	0.036
stroke	2.235	1.288–3.878	2.237	1.244–4.022	0.007
diabetes	1.909	1.405–2.595	1.462	1.042–2.051	0.028
CHD	1.425	1.182–1.719	0.941	0.750–1.179	0.596
hypertension	1.406	1.155–1.712	1.025	0.790–1.329	0.853
COPD	1.409	1.156–1.717	1.087	0.871–1.357	0.460
cerebral arteriosclerosis	1.391	1.093–1.769	1.126	0.864–1.467	0.380

1: crude hazard ratio; 2: adjusted hazard ratio (adjusted for age, systolic blood pressure, triglycerides, cigarettes per day, duration of smoking, age of starting smoking, exercise, BMI, negative affairs, family history of diseases including hypertension, stroke and cancer, and existing all kinds of diseases at baseline)

Table 4 shows that HR and 95% CI of continuous variable when divided into polytomous categories. Age, smoking index, age of starting smoking, cigarettes per day, systolic blood pressure and triglycerides had significant difference in linear trends(P < 0.05). Compared with current smokers, HR of former smokers decreased to 75.88%, following computation methods described in a previous paper [3].

**Table 4:** Hazard Ratio and 95% CI of continuous variables into 4 categories for all cause mortality

	Deaths	HR	95% CI	P	P for trend
age(year)	491				<0.001
≤ 59	75	1.000			
59–62	102	1.453	1.077–1.960	0.015	
63–66	130	2.131	1.077–2.835	<0.001	
≥ 66	184	3.405	2.599–4.462	<0.001	

smoking status*	491				
non smokers	126	1.000			
former smokers	193	1.089	0.865–1.372	0.467	
current smokers	172	1.369	1.083–1.731	0.009	
smoking index	172				<0.001
<350	34	1.000			
350–569	29	1.163	0.901–1.501	0.2457	
570–749	48	1.531	1.188–1.974	0.001	
≥ 750	61	2.069	1.642–2.606	<0.001	

age of starting smoking(year)	365				0.038
<19	101	1.000			
19–22	109	0.823	0.627–1.078	0.157	
23–27	74	0.763	0.565–1.029	0.077	
>27	81	0.720	0.537–0.966	0.028	

cigarettes per day(number)	365				<0.001
1–9	49	1.000			
10–14	32	1.093	0.700–1.707	0.694	
15–19	130	1.316	0.947–1.828	0.101	
≥ 20	154	1.831	1.327–2.527	0.001	

BMI(kg/m^2^)^Δ^	491				
<18.5	24	2.033	1.329–3.110	0.001	
18.5–23.9	188	1.000			
24–27.9	221	1.030	0.847–1.251	0.769	
≥ 28	58	1.203	0.896–1.615	0.219	

systolic blood pressure(mmHg)	491				<0.001
<120	95	1.000			
120–129	134	1.148	0.882–1.494	0.306	
130–139	89	1.509	1.129–2.017	0.005	
≥ 140	173	1.610	1.253–2.070	0.001	

triglycerides(mg/dl)	491				<0.001
<90	101	1.000			
90–122	117	1.251	0.958–1.634	0.100	
123–151	142	1.647	1.275–2.128	0.001	
≥ 152	131	1.435	1.106–1.862	0.007	

*Adjusted for age, systolic blood pressure, BMI, total cholesterol, triglycerides, regular alcohol consumption, Exercise, as well as existing disease at baseline; ^Δ^BMI was grouped by Chinese standard.

## Discussion

As estimated by WHO [1], among the 58 million deaths in the world in 2005, noncommunicable diseases accounted for 35 million, which was double the number of deaths from all communicable diseases (including HIV/AIDS, tuberculosis and malaria), maternal and perinatal conditions, and nutritional deficiencies combined. Sixteen million of the 35 million deaths occurred in people aged less than 70 years. In 2005, 53% of all deaths were among people aged 60 years and older. By 2030, this respective proportion is expected to be 62%. With aging, chronic disease will become more serious. If no action is taken, some 388 million deaths will occur in the following 10 years globally and 80 million deaths will occur in China. Just cardiac disease, stroke and diabetes will cause an economic loss of 550 billion dollars in China.

Different society, culture and economy may lead to different proportional mortality rate and disease burden in the world. The Chinese Ministry of Health reported that in China the three leading causes of death are cancers (22.94%), cerebrovascular disease (21.23%), heart disease (17.89%) in cities; and respiratory system Diseases(23.45%), cerebrovascular disease (21.27%) and cancers (20.29%) in the countryside respectively [4]. Globally, WHO's three leading death diseases are angiocardiopathy (30%), cancers (13%) and chronic respiratory disease (7%).

The objective of our study was to identify proportional mortality and risk factors of a retired military cohort. This was vital to improve the health service for Chinese retired military veterans. The three leading causes of death were found to be cancers (39.71%), angiocardiopathy and cerebrovascular disease (27.29%) and COPD (16.90%) in our cohort. The order was similar to order of death burdens in cities reported by the Chinese Ministry of Health in 2005.

Many other published cohort studies focused on a single cause of death or disease, and/or a single risk factor. For example, Eaker used data from Framingham Offspring Study and tried to identify the relationship between job stress and CHD [5]. There were few studies on the risk factors for all-cause death. Furthermore, would the risk factors observed in an elderly cohort be same to those in the general population? In China, many studies on risk factors were case-control studies. These subjects were mainly from outpatients, which could not represent the general population. In order to identify risk factors of all-cause mortality, we followed up the cohort from 1987 to 2005.

Up to now, the relationship between BMI and death has been a hot topic in the world and still has many arguments [6,7]. WHO divides BMI into four groups: underweight (BMI<18.5), normal (18.5< BMI<24.9), overweight (25< BMI<29.9) and obesity (BMI>30). Cooperative Meta-analysis Group of China Obesity Task Force [8] proposed separate criteria for Chinese adults by analyzing the data of 239972 Chinese: underweight (BMI<18.5), normal (18.5<BMI<23.9), overweight (24<BMI<27.9) and obesity (BMI>28). When our cohort was divided into four groups according to the Chinese criteria, compared with normal group, underweight group had a significant risk(HR 2.03), although HR of overweight group and obesity group was not significant (P > 0.05).

WHO reported that underweight was a risk factor of death in all age groups. Underweight was estimated to cause 3.7 million deaths in 2000. Overweight and obesity led to adverse metabolic effects on blood pressure, cholesterol, triglycerides and insulin resistance. Risks of CHD, ischemic stroke and type 2 diabetes mellitus increase steadily with increasing BMI. Raised BMI also increases the risks of cancer of the breast, colon, prostate, endometrium, kidney and gallbladder. In 2000 there were more than one billion adults worldwide overweight and at least 300 million clinically obese [8,9]. Obesity rates had raised three-fold or more since 1980 in some areas of North America, the United Kingdom, Eastern Europe, the Middle East, the Pacific Islands, Australasia and China. A new demographic transition in developing countries is producing rapid increases in BMI, particularly among the young. In China, another study got the similar result, i.e. underweight were associated with an increased risk of death in middle-aged men who had never smoked cigarettes [10].

Our result showed that risk of death raised by 0.9% while systolic blood pressure raised by 1 mmHg. When adjusted for systolic blood pressure by dividing this continuous variable into 4 categories, death risks increased significantly with increased age(P < 0.05). Porgeirsson [11] also found that age, serum total cholesterol, triglycerides, smoking and systolic blood pressure were significant independent risk factors for CHD mortality in a prospective study of 8001 randomly selected Icelandic men and 8468 women. Menotti [12] found that age, systolic blood pressure, serum cholesterol and cigarette consumption were strongly and significantly associated with all-cause mortality, coronary mortality and cardiovascular mortality. With respect to triglyceride, risk of death raised by 0.2% while triglyceride raised by 1 mg/dl. When the cohort was divided into four categories of triglyceride, death risks increased significantly with increased triglyceride (P < 0.05). In China, He Yao [13] carried out a cohort study in a machinery factory in Xi'an, China and found that triglyceride was an independent risk factor. Eberly [14] and Johansson [15] reported the similar results.

**Figure 1:**
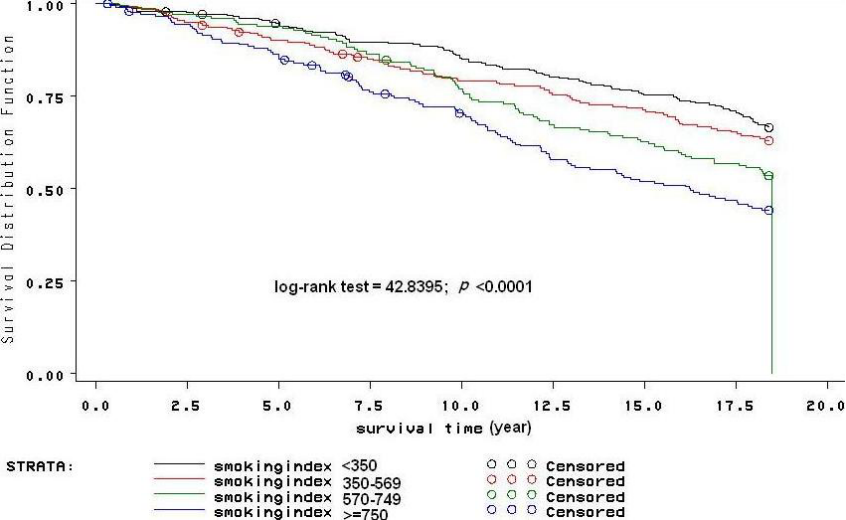
**Comparison of cumulative survival rate of different smoking index groups**. Figure 1 shows a comparison of cumulative survival rate in different smoking index groups. Cumulative survival rate decreased with increasing smoking index. Although differences between group1 (smoking index<350) and group2 (350<smoking index<569), group2 and group3 (569<smoking index<749) were not significant, there were significant differences between the other two groups (P < 0.05).

Association between smoking and deaths was reported by Doll and Peto in 1976 [16]. Our result showed that smoking was an independent risk factor for death in the cohort. At baseline, smoking rate in the cohort were 69.40%. Smoking rate was higher than those in the two census of the population in1984 and 1996(in which smoking rate of males were 61.01% and 66.94% respectively). When the subjects were divided into nonsmokers, former smokers and current smokers, current smokers' risk of all-cause death raised 0.369 when compared with nonsmokers. In current smokers, when divided into four categories according to smoking index in average, death risks increased significantly with increased smoking index (P < 0.05, as figure 1 shows). Two variables were found to be important indicators for smoking, one was age of starting smoking, and the other was cigarettes per day. Death risk increased when age of starting smoking was younger. Compared with those whose age of starting smoking were higher than 27 years, death risk increased by 28% in those whose age of starting smoking were lower than 19 years. Death risks also increased significantly with increased cigarettes per day (P < 0.05). Our results are similar to many prospective studies about smoking and related diseases in the world, finding smoking an independent risk factor for all-cause death [17-24].

As Yang reported previously [25], smoking accounted for one million deaths in China in 2000, which was 12% of total deaths. If no actions were taken, the proportion would rise to 33%, of death attributable to smoking corresponding to two million smoking-related deaths. Smoking rate of adolescents in China has risen dramatically in recent years. Age of start smoking in 2002 was four to five years earlier than that in 1984. Passive smoking is becoming more serious. As 60% of the Chinese population is passive smokers, it is urgent to develop public health measures to control the smoking problem.

According to WHO, three major risk factors for chronic disease are: unbalanced diet, physical inactivity and smoking. High blood pressure, high cholesterol, obesity, overweight, and low fruit and vegetable intake are diet-related risk factors. It is vital to control chronic disease risk factors in the population. If mortality of chronic diseases could reduce by 2% every year, 36 million lives would be saved in the following 10 years. And it will bring economic returns to the world, especially the developing countries.

There are limitations in our study. Results were based on a retired male military cohort and the representation to the general population is limited. The follow up time was short and numbers of death were small. As a result, some risk factors of all-cause mortality could not be analyzed in this cohort, e.g. drinking and diastolic blood pressure. Younger smokers could have died from smoking before retirement, and therefore were not detected in this retired cohort.

## Conclusion

The three leading causes of death were cancer, CHD and stroke disease and COPD.

Aging, cigarette smoking, high systolic blood pressure, high triglyceride, family history of hypertension, stroke and cancer, existing cases recovering from stroke, diabetes and malignant tumor, underweight, younger age of smoking were risk factors for all-cause mortality. Quitting smoking, keeping suitable levels of blood pressure, triglyceride and weight will benefit us undoubtedly.

Smoking is a major cause of death in Chinese retired military veterans. Prompt quitting of cigarette smoking should be emphasized in the control of the growing epidemic of tobacco-related diseases.

## Abbreviations

BMI: body mass index

COPD: chronic obstructive pulmonary disease

HR: hazard ratio

CI: confidence interval

CHD: coronary heart disease

## Competing interests

The author(s) declare that they have no competing interests.

## Authors' contributions

XYS contributed to the design of the present analysis of the study, the acquisition of data, carried out the data analysis, interpreted the data and drafted the manuscript.

YH contributed to the conception and design of the study and revised the manuscript for important intellectual content.

MK contributed to the acquisition of data.

BW contributed to the acquisition of data.

JYH contributed to the acquisition of data.

QLS contributed to the acquisition of data.

LZ contributed to the acquisition of data.

LSL contributed to revise the manuscript for important intellectual content.

CBCK contributed to revise the manuscript.

YPY contributed to the conception and design of the study, the acquisition of data, and revised the manuscript for important intellectual content.

All authors have read and approved the final manuscript.

## Pre-publication history

The pre-publication history for this paper can be accessed here:


